# Characteristics of the Mare-Uterine-Culture-Based Bacterial Composition Using Practical Clinical Evaluation Methods

**DOI:** 10.3390/pathogens14040357

**Published:** 2025-04-07

**Authors:** Inês B. Carvalho, Sandra Branco, Marta Laranjo, Maria Cristina Queiroga, Elisa Bettencourt

**Affiliations:** 1 MED-Mediterranean Institute for Agriculture, Environment and Development & CHANGE-Global Change and Sustainability Institute, Institute for Advanced Studies and Research, Universidade de Évora, Pólo da Mitra, 7000 Évora, Portugal; d49786@alunos.uevora.pt (I.B.C.); smbb@uevora.pt (S.B.); mlaranjo@uevora.pt (M.L.); emvb@uevora.pt (E.B.); 2 Unidade Clínica de Alter, Hospital Veterinário da Universidade de Évora, Universidade de Évora, 7440 Alter do Chão, Portugal; 3 Departamento de Medicina Veterinária, Escola de Ciências e Tecnologia, Universidade de Évora, 7000 Évora, Portugal

**Keywords:** uterine health, equine endometritis, estrus, microbiologic study, antimicrobial susceptibility test

## Abstract

Uterine health is paramount to fertility in broodmares and for the success of a breeding project, and the Lusitano breed is no exception. This study aimed to characterize the mare uterine microbiota using practical clinical evaluation methods. Mares were examined by transrectal palpation and ultrasonography, followed by the collection of samples by one of three different techniques: uterine lavage, biopsy, or swab. The results of cytology, histology, microbiology, and antimicrobial susceptibility testing were recorded, and statistical analyses were performed. Inflammation was present in 42.2% of the mares and positive culture in 65.4%. *Escherichia coli* and *Streptococcus* spp. were the most isolated microorganisms. The most efficient antimicrobials were gentamicin, trimethoprim-sulfamethoxazole, and enrofloxacin and resistance was detected mainly for doxycycline, penicillin, and ceftiofur. The phase of the cycle was significantly associated with the presence of inflammation (*p* = 0.0308). The isolation of Gram-positive or Gram-negative bacteria correlated to the microbiological isolation by primoculture/enrichment processes (*p* = 0.0183). This was a routine standard breeding evaluation of broodmares in the management of a stud farm, hence displaying the characteristics of a field study. The antimicrobial resistance findings reinforce the importance of performing microbiology and susceptibility tests, even under field conditions, to maximize targeted antimicrobial therapy efficiency and minimize the worldwide problem of antimicrobial resistance, promoting antimicrobial stewardship.

## 1. Introduction

Uterine health in the broodmare is evaluated in practice by clinical and ultrasonographic examinations, in association with cytology, microbiology, and histopathology analysis. Equine endometritis continues to be a major cause of infertility in mares and its prevalence can be quite high in a broodmare population [1].

The different sample collection methods, namely uterine swab, uterine flush (UF), and biopsy, have advantages and disadvantages. A culture from UF is more sensitive because the saline contacts with a larger endometrial surface area, but the same is not necessarily true for cytology. The fluid may not expose the degree of inflammation in the endometrium because centrifugation can result in disruption of cell walls [2], hence showing the poor preservation of the cells and presence of more debris [1]. Regarding swabs, cytobrush samples can provide an excellent sample for cytology [3]. Nevertheless, positive cytology is observed in a greater number of smears obtained by biopsy than in those obtained by low-volume lavage (LVL) or swabs [4]. Biopsy is also more sensitive for microbiological examination than an endometrial swab [5]. However, it is more costly, it is invasive, and there is a perceived negative effect on fertility if breeding occurs in the same cycle [1]. The histological presence of polymorphonuclear neutrophils (PMNs) in the endometrium is the most reliable diagnosis of endometritis [5]; hence, endometrial biopsy is considered the gold standard technique [1,5]. Furthermore, endometrial biopsy is the only method to diagnose mares with chronic endometritis and correlates with the prognosis for a mare to carry a foal to term [1].

Different microorganisms are described as common bacteria associated with equine endometritis, such as *Escherichia coli*, *Staphylococcus* spp., and *Streptococcus* spp. [6]. In vitro antimicrobial susceptibility testing, prior to antimicrobial therapy, is crucial concerning infectious endometritis to determine the best antimicrobial to successfully overcome the infection. The careless preventive administration of “first choice” antimicrobials has resulted in the development of resistant strains to broad-spectrum antimicrobials from several microorganisms [7]. Antimicrobial resistance (AMR) refers to microorganisms’ capacity to withstand the impact of an antimicrobial to which they were previously susceptible, allowing germs to survive and thrive. It is now considered one of the most serious challenges to public health [8]. In equine reproduction, the literature indicates that bacteria with AMR in the equine uterus and vagina have been reported worldwide. In broodmares, AMR may arise from local and systemic antimicrobial use, but also from semen extenders used in artificial insemination (AI) [9].

The aims of this study were to (1) characterize the microbiome of the mare uterus in the estrus and the diestrus using different sampling methods, (2) study the effect of the enrichment procedure on the accuracy of bacterial identification, (3) evaluate the inflammatory uterine response versus bacterial isolation, and (4) assess bacterial susceptibility to a set of antimicrobials currently used to treat endometritis in mares in field conditions.

## 2. Materials and Methods

### 2.1. Animals

Samples (n = 106) were collected in a herd of Lusitano mares, aged 4 to 24 years old (the mean age of mares in this study was 12.6 years), between 2021 and 2022, at a stud farm in Portugal serving approximately 60 mares by artificial insemination (AI) per breeding season. Based on their reproductive status, mares were divided into five groups (Table 1).

The mares are client-owned; hence, not all procedures were performed in all mares. All the mares available for the breeding season of the stud farm were included in the current study.

### 2.2. Reproductive Examination and Sample Collection

Mares were examined restrained in stocks and a comprehensive reproductive assessment was performed before collection of samples, including transrectal manual and ultrasonographic evaluation. The following parameters were recorded: presence and maximal height (cm) of intrauterine fluid measured in the uterine body, endometrial oedema (score—0: no oedema, 1: mild oedema, 2: moderate oedema, 3: severe oedema), and number and size (mm) of dominant follicle(s) [10].

After transrectal examination, the collection of samples was performed as described in previous studies [2,3,9,11,12]. For each mare, the tail was wrapped and elevated, and vulva and perineum were scrubbed with chlorhexidine solution and dried with a paper towel.

For uterine lavage (UL), a sterile uterine Foley catheter lavage system was passed through the vagina and cervix, conducted by a veterinarian with a sterile long sleeve glove. The balloon on the catheter was filled with air, hence fixing the catheter inside the uterus. A volume of 500–1000 mL of sterile physiological saline was infused in the uterus and drained back to its recipient by gravity flow. The volume recovered was recorded and the sample was then manipulated in a laminar flow cabinet, subdividing it into two different 50 mL sterile Falcon tubes, one for culture and one for cytological analysis.

For collection of samples by uterine swabs, double-guarded swabs were manually inserted in the uterus by a veterinarian wearing a sterile long sleeve glove after preparation of the mare in a similar fashion as described before. The swab was extended, gently rolled for approximately 30 s, and then retracted through the guard. It was sent to the laboratory at 4 °C within 24 h.

For collection of biopsy samples, the same technique of asepsis and insertion in the uterus was performed but with a sterilized biopsy punch instrument. The sleeved arm was withdrawn from the vagina and introduced into the rectum to guide the forceps and place endometrial mucosa within it, obtaining a small sample of the endometrium. The sample was then manipulated in a laminar flow cabinet, subdividing it in two pieces: one was placed in a sterile Falcon tube for microbiology; other was smeared on to the slide for cytology and then fixed in 10% buffered formalin for histology.

Overall, 59 samples were collected by uterine lavage, 34 samples by uterine biopsy, and 13 samples by uterine swab.

### 2.3. Cytology

For cytology, the sample in one Falcon tube was immediately centrifuged at 800× *g* for 10 min. The supernatant was decanted, and a swab was used to smear the pellet on a microscope glass slide.

The swab was rolled on a slide, which was allowed to air-dry. Then, the smear was fixed immediately with methanol and stained with MG Quick (Bio-Optica Milano SpA, Milan, Italy). The slide was examined with light microscopy under oil immersion (×1000). Presence of inflammation was considered if the amount of PMNs was >5% of the total number of cells. Guidelines for the degree of inflammation according to the percentage of neutrophils were as follows: 5–15% mild inflammation, 15–30% moderate inflammation, and >30% severe inflammation [13].

### 2.4. Microbiology (Culture)

For microbiology analyses, the lavage was centrifuged at 5000× *g* for 10 min at 4 °C. Supernatant was discarded and the remaining liquid was cultured with a disposable loop (approximately 10 μL). Samples (10 μL of centrifuged uterine lavages, swabs, and biopsy) were plated onto MacConkey agar (Oxoid, CM0007, Basingstoke, Hampshire, UK) and Columbia blood agar (BA) (BioMérieux, 43041, Marcy-l’Étoile, France) and inoculated in Tryptose phosphate broth (Scharlau, 02-199-500, Barcelona, Spain) for enrichment. All cultures were incubated at 37 °C for 24 h. If no growth was observed, the plates were reincubated for further 24 h in the same conditions and the enriched culture was plated onto MacConkey agar and Columbia blood agar, followed by 24 h incubation at 37 °C.

Primary identification through morphological and biochemical characteristics, namely, colony morphology, Gram staining, and catalase and oxidase reaction, according to Markey et al. [14], was performed followed by identification to the species level by automated compact system VITEK 2 (BioMérieux, Marcy-l’Étoile, France). Biochemical identification was confirmed by 16S rRNA gene sequencing whenever necessary. For the isolates where biochemical identification was not conclusive or the authors were uncertain of a correct result, total DNA was extracted using a rapid DNA extraction protocol [15] and the 16S rRNA gene amplified and sequenced using primers Y1 and Y3 [16,17]. 16S rRNA gene sequences were analyzed with MEGA software version 11.0.13 [18]. In these cases, molecular identification prevailed.

Mares were excluded from the study if more than three different bacterial colonies were present; hence, we considered contamination of the sample in question might have occurred throughout the process.

All isolates were then tested for antimicrobial susceptibility by one of two methods: disk diffusion [19] and Vitek 2 (BioMérieux, Marcy-l’Étoile, France). Bacterial growth inhibition was evaluated and the results categorized as resistant or non-resistant according to the guidelines of the Clinical and Laboratory Standards Institute (CLSI) [19] and the European Committee on Antimicrobial Susceptibility Testing (EUCAST) [20]. Tested antimicrobials were selected based on commonly used antimicrobials in equine practice: gentamicin, trimethoprim-sulfamethoxazole, penicillin, tetracycline, ceftiofur, enrofloxacin, and doxycycline. *Staphylococcus aureus* ATCC 25923 and *Escherichia coli* ATCC 25922 reference strains were used as control strains.

Antimicrobial susceptibility tests were performed on a total of 105 isolates. Contrarily to the current approach in sensitivity testing for tetracyclines, guidelines in horses indicate not to test tetracycline as a surrogate for doxycycline and minocycline [19]. The sensitivity of trimethoprim-sulfamethoxazole against *Enterococcus* spp. was not determined because its interaction is uncertain and therefore it is not possible to predict clinical outcome [20]. According to EUCAST, if no interpretative breakpoints are available for certain antimicrobials and microorganisms, they can be reported resistant without further testing when considering the correct therapy to apply [20]. However, for the purpose of the current study, when a certain antimicrobial was not tested for an isolate, it was not considered for the calculation of resistance.

### 2.5. Histopathology

The samples were fixed in 10% neutral-buffered formalin and processed for examination by standard light microscopy techniques. Histologic sections of the endometrial biopsy specimen were examined for the presence of inflammatory and degenerative changes. Inflammation was recognized by the accumulation of PMNs in the endometrial tissue. Degenerative changes detected in biopsy evaluation included cystic dilation of glands and glandular necrosis. Also, depositions of fibroblasts around endometrial glands were important findings to note. Following these observations, the endometrium was classified on a grading ranging from I to III, according to the degree of endometrial changes, which allowed us to determine the prognosis for a mare to carry a foal to term. Grade I was essentially normal, with minimal inflammation or fibrosis; Grade II was often divided into subcategories IIA and IIB, encompassing mild to moderate pathologic conditions, and Grade III endometrium included severe inflammatory and/or fibrotic changes [21].

### 2.6. Statistical Analysis

The software Statistica 12 (©StatSoft, Inc., Tulsa, OK, USA, 1984–2014) was used for the statistical analyses. All probability values were computed using Pearson’s Chi-Square test of association; *p* < 0.05 was considered significant.

Statistical analyses were conducted to tentatively answer the following questions: (1) Is there a relationship between the results of microbiology and inflammation? (2) Does the differential presence of Gram-positive or -negative bacteria affect the presence of inflammation? (3) Is primoculture and enrichment process important for specific isolation of Gram-positive and -negative bacteria? (4) Does the phase of the cycle influence the presence of inflammation? (5) Does the age of the mare affect the presence of inflammation or positive culture? (6) Is the method of collection important to enhance detection of inflammation/positive culture?

Prevalence rates of AMR were calculated for each bacterial species.

## 3. Results

From among the samples sent to cytological or histological analysis (n = 83), 42.2% of the mares had some degree of inflammation. Concerning microbiological analysis (n = 104), 65.4% presented positive cultures (Table 2). A total of 124 isolates were obtained from the 68 positive mares.

**Figure 1:**
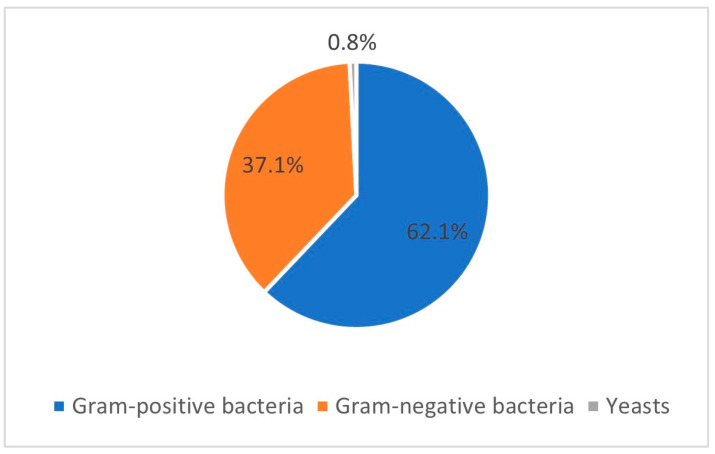
Prevalence of Gram-positive bacteria, Gram-negative bacteria, and yeasts regarding the 124 isolates obtained from the 68 positive mares included in the study.

*E. coli* and *Streptococcus* spp. were the most isolated microorganisms in this group of mares. Yeasts were observed in only one mare. Gram-positive and -negative bacteria were observed in 77 and 46 isolates, respectively (Figure 1).

**Figure 2:**
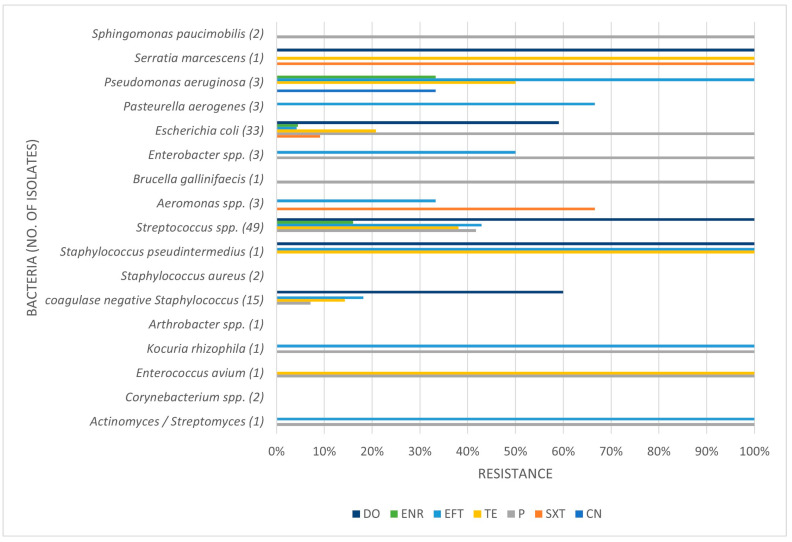
Antimicrobial resistance profile regarding all isolates. CN: gentamicin; SXT: trimethoprim-sulfamethoxazole; P: penicillin; TE: tetracycline; EFT: ceftiofur; ENR: enrofloxacin; DO: doxycycline.

**Figure 3:**
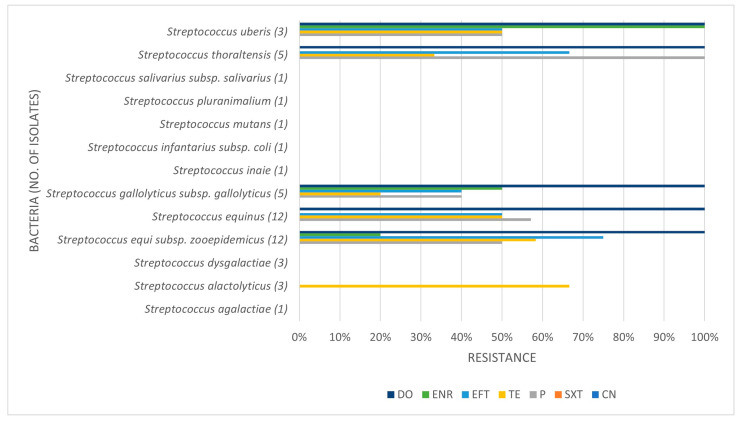
Antimicrobial resistance profile regarding *Streptococcus* spp. CN: gentamicin; SXT: trimethoprim-sulfamethoxazole; P: penicillin; TE: tetracycline; EFT: ceftiofur; ENR: enrofloxacin; DO: doxycycline.

**Figure 4:**
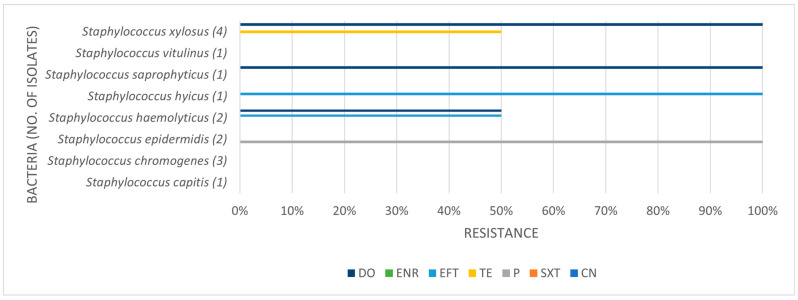
Antimicrobial resistance profile regarding coagulase negative staphylococci. CN: gentamicin; SXT: trimethoprim-sulfamethoxazole; P: penicillin; TE: tetracycline; EFT: ceftiofur; ENR: enrofloxacin; DO: doxycycline.

**Figure 5:**
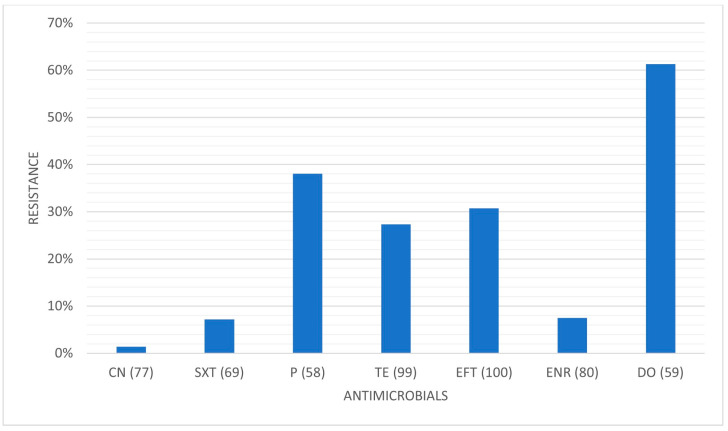
Average antimicrobial resistance for each studied antimicrobial. CN: gentamicin; SXT: trimethoprim-sulfamethoxazole; P: penicillin; TE: tetracycline; EFT: ceftiofur; ENR: enrofloxacin; DO: doxycycline. (x): number of isolates tested for that specific antimicrobial.

Table 3, Table 4 and Table 5 present the prevalence of isolated pathogens in this study and the results of antimicrobial susceptibility testing. Figure 2, Figure 3, Figure 4 and Figure 5 show the average antimicrobial resistance for each studied antimicrobial. When results from antimicrobial susceptibility were evaluated, the most efficient antimicrobials were gentamicin, trimethoprim-sulfamethoxazole, and enrofloxacin. All Gram-positive bacteria evaluated were susceptible to the trimethoprim-sulfamethoxazol association. It was revealed that a high proportion of the tested bacteria showed resistance to broad-spectrum doxycycline and ceftiofur, and to penicillin. For example, numerous *Streptococcus* spp. were resistant to penicillin and ceftiofur, and all showed resistance to doxycycline. However, *E. coli* showed low resistance (less than <10%) to ceftiofur and total susceptibility to gentamicin.

Some isolates were multidrug-resistant (MDR) strains, namely seven *Str. equi zooepidemicus*, four *Str. equinus*, two *Str. gallolyticus gallolyticus*, two *Str. thoraltensis*, one *Str. uberis*, one *S. pseudintermedius*, one *E. coli*, one *Pseudomonas aeruginosa*, and one *Serratia marcescens.*

The results of culture and inflammation were not significantly associated (*p* = 0.5687). A total of 29.4% of mares with positive inflammation (cytology and histopathology) had negative culture results, and 45.6% of mares with positive culture had negative cytology results.

The presence of inflammation was not associated with the isolation of Gram-positive or Gram-negative bacteria (*p* = 0.1687). However, the isolation of Gram-positive or Gram-negative bacteria was associated with microbiological isolation by primoculture/enrichment processes (*p* = 0.0183), showing that more microorganisms were isolated on primoculture. In fact, concerning bacterial positive results, 63.2% of the samples exhibited microbial growth on primoculture, followed by 36.8% that required the enrichment procedure.

The phase of the cycle was not associated with the culture results (*p* = 0.1737) but was significantly associated with the presence of inflammation (*p* = 0.0308), showing that mares in foal heat displayed more inflammation and, conversely, mares in transition/inactive were less likely with uterine inflammation.

There was no significant association between age and inflammation (*p* = 0.1604) or culture results (*p* = 0.0952).

The method of collection was not associated with the presence of the inflammation (*p* = 0.2962) or infection (*p* = 0.4458).

## 4. Discussion

In this study, 106 mares were analyzed. As the mares are client-owned, not all procedures were performed in all cases. For the performance of UL, the authors opted to infuse a larger amount of saline than that generally used for LVL because it was a study made in clinical practice and so had to mimic the usual treatment performed before AI. Therefore, we respected the normal management of the stud farm herd aiming at maximizing the breeding efficiency, using a sampling technique that could be simultaneously part of the treatment.

About 42% of the overall mares sampled presented inflammation, a value that was slightly lower that the 52% described in another study with multiple collection techniques [12]. When the sample was obtained using biopsy, which is considered the accredited gold standard [4,21], the results showed that 33% of those mares had cytological inflammatory signs. These results were within the spectrum of values on endometrial biopsy across studies, ranging from 15% to 35.5% [4,21,22]. Differences amongst studies are not a surprise given (1) the nature of a stud farm versus a referral hospital population; (2) the multiple collection methods versus the exclusive use of the gold standard biopsy for the diagnosis of endometritis; (3) the possible presence of degenerated neutrophils arising from the use of uterine lavage in some studies; (4) the different interpretation of cytology results, namely the threshold for the presence of inflammation; and (5) the different horse populations being compared. In fact, the use of different sampling techniques and analysis of results across studies, namely different cytological thresholds, hindered comparability of results. These facts highlight the need for the standardization of methodologies [12].

Regarding microbiological results, approximately 65% of the mares showed microbial growth, which, despite the different sampling techniques across studies, was within the range of values previously reported [7,10]. *E. coli* and *Streptococcus* spp. were the most isolated microorganisms in the mares in this study, which was also in accordance with the literature [1,2,7,23,24,25,26], and it is well known that these bacteria are involved in the reproduction disorders of females [7].

Resistance to antimicrobials has been commonly described and poses a serious health problem. It is worth noting the relevant AMR patterns obtained towards those seven antimicrobials widely used in horses. The main reason for the decrease in antimicrobial efficacy has been associated with their inappropriate application by either over-prescription, overuse, or the inadequate following of the antimicrobial course [6]. Actually, most microorganisms have the capability of developing resistance to at least some antimicrobial agents. Microorganisms are living organisms that adapt over time; hence, it is the natural process of bacteria to develop drug resistance. The mechanisms of resistance can be intrinsic or acquired [27]. Acquired resistance is due to the selective pressure in which the conditions, for example, the incorrect use of an antimicrobial, allow the survival and proliferation of resistant organisms with newly characteristics, rapidly overtaking the microbial population as the dominant form [8]. Healthy farm animals can be reservoirs of AMR as recent studies have elucidated the troublesome situation of the antimicrobial treatment of animals in a stable influencing the resistance patterns of both treated as well as untreated animals housed in the same stable [28]. In addition, there are reports of broilers acting as reservoirs of AMR for a drug forbidden in poultry [29]. In fact, healthy farm animals have been found to act as a reservoir of extended-spectrum β-lactamase (ESBL)-producing *E. coli*, even in groups that have not received any treatment. Ceftiofur and its metabolites were present in the excrements (urine and feces) of the treated animals and could be found in the dust and aerosol of the stable and, even more concerningly, ESBL-carrying *E. coli* was also detected in the aerosol. Over eighty percent of the applied ceftiofur dose was excreted as an antimicrobial active form due to the presence of the intact β-lactam-ring, hence retaining microbicidal activity. These could be the trigger for the resistance pattern of initially untreated animals [28]. The existence of resistant genes in the microbiota of broilers may pose a human health hazard since these bacteria may represent a reservoir of resistance genes for pathogens causing disease in humans and other animals [29].

A high proportion of the tested bacteria showed resistance to the broad-spectrum antibiotics doxycycline and ceftiofur and the commonly used penicillin. Focusing on penicillin, 38.1% of the isolates were shown to be resistant. Despite the high level of resistance found here, it is worth pointing out that *Staphylococcus* spp. showed good susceptibility to this antimicrobial. Bacterial resistance to penicillin has progressively increased over the years. The reported percentage of sensitivity nowadays ranges from 0% to 38.3%, and Gram-negative bacteria are more resistant to penicillin than Gram-positive bacteria [6]. The main reason for this is that Gram-negative species contain an outer membrane, which acts as a selective barrier, blocking the penetration of penicillin. However, acquired specific genes that encode for penicillinases (also known as beta-lactamases), enzymes capable of inactivating penicillin by hydrolysis of the beta-lactam ring in its structure, are present in Gram-negative bacteria, although Gram-positive bacteria can also produce them. As such, this antibiotic has a narrower spectrum of activity, namely used against Gram-positive bacteria [30]. Worryingly, we found almost 42% of *Streptococcus* spp. to be resistant to penicillin. In our study, Gram-negative bacteria were not tested for penicillin resistance.

Concerning ceftiofur, a third-generation cephalosporin antimicrobial, the production of extended-spectrum β-lactamases (ESBL) is the most important resistance mechanism and is most commonly used by Gram-negative bacteria. Gram-negative bacteria that frequently present these β-lactamases are *Aeromonas* spp., *Pseudomonas* spp., and members of the *Enterobacteriaceae* family [27]. In fact, our study always detected some degree of resistance from these microorganisms to ceftiofur. Furthermore, it was the third antimicrobial with higher level of resistance, about 30%, which leads us to suggest that it might have been frequently used as a first-choice antimicrobial.

Regarding enrofloxacin, there were some, although few, resistant isolates of *Streptococcus* spp., *E. coli*, and *Pseudomonas aeruginosa*, which may indicate it is also being used as a first-choice antimicrobial.

The World Health Organization states clear evidence of adverse human health consequences due to resistant organisms resulting from the non-human usage of antimicrobials and, for this reason, has appointed a list of antimicrobials that are considered critically important for humans. In this list, amongst the antimicrobials classified as highest-priority critically important antimicrobials (HPCIAs) are third-, fourth-, and fifth-generation cephalosporins and quinolones, in which ceftiofur (although exclusively for animal use) and enrofloxacin are included [31]. In our study, a high proportion of the tested bacteria showed resistance to the broad-spectrum antibiotic ceftiofur. Moreover, although enrofloxacin was found to be one of the most efficient antimicrobials in vitro, some isolates showed resistance, so its use in animals should be avoided. As aforementioned, the careless preventive administration of “first choice” antimicrobials has resulted in the development of resistance to antimicrobials from several microorganisms. It is therefore important to use antimicrobials appropriately, namely with the use of narrow-spectrum antimicrobials as “first choice”, and whenever possible oriented by susceptibility analysis [7]. AMR in the microbiota of the mare’s reproductive tract has been reported in many countries and involves resistance against several antimicrobial agents. The horizontal gene transfer (HGT) of antimicrobial resistance is possible through conjugation, transduction, and transformation. Even dead bacteria can pass on resistance genes to other bacteria by this method. Therefore, it is advisable to avoid the use of antimicrobials whenever possible because this poses one of the greatest challenges facing humankind in the modern world [9].

Tetracycline antibiotics, such as the least efficient antibiotic in our study (doxycycline), block protein synthesis by binding reversibly to the 30S ribosomal subunit. Tetracycline resistance is a textbook example of efflux-mediated resistance, in which efflux pumps use proton exchange as a source of energy to extrude tetracyclines. With these being hydrophilic drugs, Gram-negative bacteria are intrinsically less permeable due to the lipopolysaccharide layer in their outer membrane, which creates a permeability shield [8]. Doxycycline was the antibiotic with the most resistance level in our study. Most *E. coli* isolates showed resistance to doxycycline, as did all *Streptococcus* spp., the latter fact suggesting that this drug might have been overused in practice. In horses, guidelines indicate not to test tetracycline as a surrogate for doxycycline, contrary to what is generally advocated. Indeed, our in vitro results showed significantly different resistance levels for both antimicrobials, highlighting the importance of preventing the misuse of doxycycline in an isolate that tested susceptible to tetracycline.

Unfortunately, many *Streptococcus* spp., common uterine pathogens found across studies, were resistant to these routinely applied antimicrobials, which highlights the problem of AMR. Thus, studying the bacterial susceptibility to these drugs is relevant for clinical use and adequate treatment. The most efficient antibiotic was gentamicin, followed by trimethoprim-sulfamethoxazole and enrofloxacin. Furthermore, all Gram-positive bacteria evaluated were susceptible to gentamicin and trimethoprim-sulfamethoxazole. Regarding gentamicin, in general, this finding was in agreement with other studies [6]. All isolates were sensitive to gentamicin, except *Pseudomonas aeruginosa*, which showed about 33% of resistance. Focusing on *P. aeruginosa* infections, this microorganism is intrinsically resistant to many antimicrobials, such as sulphonamides, ampicillin, first- and second-generation cephalosporins, tetracycline, and others [27]. However, one other study showed that gentamicin is efficient against it, and also showed that this microorganism is highly sensitive to trimethoprim-sulphonamide, a finding in accordance with our study [6]. In fact, looking at the association of trimethoprim-sulfamethoxazole, we showed that all Gram-positive and most Gram-negative isolates were susceptible. In clinical practice, the intrauterine use of some antimicrobials is a great asset to achieve local bactericidal/bacteriostatic concentrations but challenging. For example, gentamicin is very acidic and requires buffering with sodium bicarbonate to a more neutral pH before infusion in the uterus. In contrast, enrofloxacin has a basic pH and when buffered to a more neutral pH is reported to lose efficiency. The unbuffered use of enrofloxacin can be very irritant to the endometrium; therefore, if the susceptibility pattern suggests its use, systemic treatment is recommended [10].

Finally, the antimicrobial resistance findings in the present study reinforce the importance of performing a microbiological study combined with sensitivity tests to better determine the actual responsible microorganism of endometritis in the mare and the most appropriate therapy. In light of all these findings, the authors would like to (1) highlight that drugs listed as critically important for human medicine, as quinolones and third-generation cephalosporins, should not be used as first choice antimicrobials, which apparently might be happening, given the resistance levels found; and (2) suggest, as first-choice antimicrobials, the use of the association of gentamicin with trimethoprim-sulfamethoxazole if and when susceptibility testing is not available.

The results showed that culture and inflammation were not significantly associated. Mares showing inflammation with negative microbial culture (29.4%) had possibly a non-infectious endometritis induced by an unresolved inflammation or an infection with bacteria protected in biofilms that impaired the success of the bacterial culture [6]. On the other hand, mares lacking inflammation but with a positive culture (45.6%) could have been the result of the contamination of the sample or the natural microbiome of the uterus [32]. In our opinion, knowing that the rigorously aseptic collection of samples was performed, such positive cultures could have been the result of the natural microbiome of the uterus, with it being nowadays recognized that the uterus is not sterile as once thought, following the publication of the Human Microbiome Project in 2007 [32]. Furthermore, it is known that *Streptococcus* spp. are normal components of the vaginal microbiota of healthy mares [24], and therefore, it is no wonder that streptococci are part of the transient microbiota of the uterus. Riddle et al. considered both endometrial cytology and culture as diagnostic for identifying mares with endometritis as pregnancy rates were decreased when either test was positive. The isolation of microorganisms resulted in lower pregnancy rates, regardless of cytological findings, but endometrial cytology identified twice as many mares with endometritis as did endometrial culture [25]. Moreover, beta-haemolytic streptococci, *E. coli* and *Pseudomonas*, known uterine pathogens, represented more than 60% of the isolates recovered in that study. In contrast, a more recent study stated that culture is unreliable as a single diagnostic criterion as few mares with endometritis had positive culture results [1]. The fact that our results revealed that mares with inflammation may not show infection and others with detected positive culture may not display inflammation reinforces the need for always associating cytological and microbiological information. The real need for using antimicrobials in the treatment of endometritis in the mare should be careful evaluated and the possibility of use alternative treatments must be considered [33].

Association between the culture process and the isolation of Gram-positive or Gram-negative bacteria showed that more microorganisms are isolated on primoculture. More than half of the bacteria grew after primoculture, but the enrichment process was important to fully detect a positive microbiology, because about forty percent of the isolates were only possible to detect due to the enrichment. The enrichment step in the routine bacteriological examination increases the isolation rate and recovers additional isolates from those samples that are negative after direct smearing [24]. A previous study showed that Gram-negative bacteria grow well with no pre-enrichment whilst Gram-positive bacteria often require enrichment. In fact, the authors noticed, specifically, the isolation of *Streptococcus* spp., *Staphylococcus* spp., and *Bacillus* spp. to be considerably increased after enrichment. It is noteworthy that the enrichment process in traditional microbiology can be a major advantage towards the appropriate diagnosis and treatment of mares. As always, using an appropriate sampling technique is essential to minimize the contamination of the samples because other microorganisms, such as the natural microbiota of the vagina, can grow and do not reflect the uterine condition [24].

The phase of the cycle is significantly associated with the presence of inflammation (*p* = 0.0308), showing that mares in foal heat display more inflammation and inversely, mares in transition/inactive are less likely with uterine inflammation. However, the phase of the cycle does not correlate to the presence of infection. In the estrus, a small amount of PMNs is considered a normal finding in healthy mares because estrogen dominance promotes neutrophil migration more than progesterone dominance. Possibly, different thresholds of inflammation should be used to evaluate the results obtained during different sampling periods [33].

In this study, the age of the mare did not correlate to the presence of a positive culture or inflammation. This was in contrast to other studies and in-practice experience that states that mares increase their susceptibility to post-breeding endometritis as they get older due to age being a factor in the progression of degenerative endometrial changes, the hence-decreased endometrial quality [34], and susceptibility to infection. Moreover, older barren and foaling mares may need to be treated more frequently than younger and maiden mares, as well as typically requiring more matings per conception [35].

Lastly, the method of collection did not interfere with the presence of inflammation or infection. Similarly, a study from 2022 did not find difference in the sample collection technique for the cytological assessment [12], but other authors consider biopsy as the best method to detect inflammation [4]. In our study, the described poor preservation of cells and more debris in UL [1] did not affect the detection of inflammation. This could perhaps be explained by the use of a larger volume of saline for the lavage than usually used. Sensitivity was not affected, because this larger volume was centrifuged prior to culture. Regarding microbiology, one study showed that uterine lavages with either high or low volumes of fluid increased the sensitivity of the bacteriological identification. In fact, flush culture doubled the ability to detect infected mares based on culture alone [2]. Similarly to the technique of our study, macro-flushing samples was a good technique to isolate microorganisms [24]. This enlightens the advantage of the flush to assess the entire endometrium. On the other hand, different studies have reported different findings. One study found out that the double-guarded cytobrush is more sensitive than the low-volume flush for microbiology [12], but other authors stated that a bacterial culture obtained from swabs showed a lower sensitivity than that obtained by biopsy [23]. In our study, a higher number of positive cultures was obtained by biopsy, followed by UL and lastly by swabs. The standard sampling method is uterine biopsy; however, as most of these mares were in the estrus, and could be inseminated as part of the reproductive program of the stud farm, it was not possible to perform biopsies on all animals. Uterine lavage is an excellent alternative for culture, and regarding biofilms, we believe the washing technique may be the best to collect the samples as this procedure most probably disrupts the biofilms, allowing the recovery of sessile bacteria.

The main limitations of this study were related to the fact that it was a study in clinical practice; hence, the sample collection was not standardized as it could have been if it was a pilot study. The mares studied were included in an artificial insemination program, which limited the possibility of standardizing the sampling and collection methodologies that could have been applied. However, the fact that this study was realized in field practical conditions is relevant for veterinarians and stakeholders, revealing the applicability of the collection and evaluation methods of the diagnosis of endometritis in the mare.

In future studies, it will be desirable to evaluate only mares in the same estrous cycle phase with only one collection methodology and applying biomarkers of uterine inflammation, such as metagenomic analysis, to improve the sensibility of the diagnosis of both inflammation and microbiological evaluations.

## 5. Conclusions

Uterine health is paramount to fertility in broodmares. This study was the result of the routine standard breeding evaluation of Lusitano broodmares in the management of a stud farm, hence displaying the characteristics of a field study. In response to the objectives aforementioned, (1) the stage of the cycle did not influence the results of cytology and/or culture, (2) the enrichment process improved the diagnosis ability, (3) there was no significant association between inflammation and positive culture, and (4) antimicrobial susceptibility tests are crucial to determine the best treatment for infectious endometritis.

Additionally, as per our study, under field conditions, UL with a high volume of fluid is a good collection method for laboratory evaluations, and it simultaneously acts as part of therapy. It seems to be a good management tool under field conditions to maximize the reproductive efficiency of broodmares, particularly those prone to endometritis. According to our results, we may suggest, as first-choice antimicrobials, the use of the association of gentamicin with trimethoprim-sulfamethoxazole if and when susceptibility testing is not available.

## Figures and Tables

**Table 1:** Demographic information of mares included in the study.

Group	Reproductive Status	No. of Animals
**Estrus mares**	Mares with endometrial oedema and a follicle >25 mm diameter by ultrasound, and a flaccid uterus	61
**Foal heat mares**	Mares in estrus in the first cycle immediately after parturition	11
**Diestrus mares**	Mares with visual identification of corpus luteum (CL) by ultrasound, lack of uterine oedema and presence of uterine tone	16
**Transition mares**	Mares with multifollicular ovaries, shaped as a ‘grape cluster’	11
**Inactive mares**	Mares with small inactive ovaries	7
**TOTAL**		106

**Table 2:** Frequency of positive results per collection method.

Sampling Method	Inflammation			Culture		
	Positive	Negative	Frequency (%)	Positive	Negative	Frequency (%)
**LVL**	25/52	27/52	48.1%	37/58	21/58	63.8%
**Biopsy**	10/30	20/30	33.3%	24/33	9/33	72.7%
**Swab**	0/1	1/1	0%	7/13	6/13	53.9%
**Total**	35/83	48/83		68/104	36/104	
**Frequency**	42.2%			65.4%		

**Table 3:** Prevalences of bacterial species isolated in this study and antimicrobial resistance (number of resistant isolates out of the number of tested isolates).

Isolates	Frequency		Antimicrobial Resistance						
	n	%	CN	SXT	P	TE	EFT	ENR	DO
**Gram-positive**									
***Actinomyces*** **/*Streptomyces***	1	0.8%	0/1	0/1	1/1	0/1	1/1	0/1	−
***Corynebacterium* spp. ^1^**	2	1.6%	0/1	0/1	0/1	0/1	0/1	0/1	−
***Enterococcus avium***	1	0.8%	0/1	-	1/1	1/1	0/1	−	0/1
***Kocuria rhizophila***	1	0.8%	0/1	0/1	1/1	0/1	1/1	0/1	0/1
***Arthrobacter* sp.**	1	0.8%	0/1	0/1	0/1	0/1	0/1	0/1	−
**Coagulase negative** ***Staphylococcus***	15	12.1%	0/14	0/11	1/14	2/14	2/11	0/13	6/10
***Staphylococcus aureus***	2	1.6%	0/2	0/2	0/2	0/2	0/2	0/2	0/2
***Staphylococcus pseudintermedius***	1	0.8%	0/1	0/1	0/1	1/1	1/1	0/1	1/1
***Streptococcus* spp.**	49	39.5%	0/20	0/20	15/36	16/42	18/42	4/25	17/17
**Gram-negative**									
***Aeromonas* spp. ^2^**	3	2.4%	0/3	2/3	−	0/3	1/3	0/1	0/1
***Brucella gallinifaecis***	1	0.8%	0/1	0/1	−	0/1	0/1	0/1	−
***Enterobacter* spp. ^3^**	3	2.4%	0/1	0/1	−	0/2	1/2	0/2	01
***Escherichia coli* ^4^**	33	26.8%	0/24	2/22	−	5/ 24	1/24	1/22	13/ 22
***Pasteurella aerogenes***	3	2.4%	−	−	−	−	2/3	0/3	−
***Pseudomonas aeruginosa***	3	2.4%	1/3	0/1	−	1/2	3/3	1/3	0/1
***Serratia marcescens***	1	0.8%	0/1	1/1	−	1/1	0/1	0/1	1/1
***Sphingomonas paucimobilis***	2	1.6%	0/2	0/2	−	0/2	0/2	0/2	0/1

CN: gentamicin; SXT: trimethoprim-sulfamethoxazole; P: penicillin; TE: tetracycline; EFT: ceftiofur; ENR: enrofloxacin; DO: doxycycline. −: no result available. ^1^ One isolate was *Corynebacterium uterequi*. ^2^ Two isolates were *Aeromonas media*. ^3^ One isolate was *Enterobacter hormaechei*. ^4^ Three isolates were haemolytic *E. coli*.

**Table 4:** Prevalence of *Streptococcus* spp. isolated in this study and antimicrobial resistance (number of resistant isolates out of the number of tested isolates).

Isolates	Frequency		Antimicrobials Resistance						
	n	%	CN	SXT	P	TE	EFT	ENR	DO
***Streptococcus agalactiae***	1	0.8%	−	−	0/1	0/1	0/1	−	−
***Streptococcus alactolyticus***	3	2.4%	−	0/1	0/3	2/3	0/3	−	−
***Streptococcus dysgalactiae* ^5^**	3	2.4%	−	−	0/2	0/3	0/3	0/1	−
***Streptococcus equi* subsp. *zooepidemicus***	12	9.8%	0/10	0/8	6/12	7/12	9/12	2/10	7/7
***Streptococcus equinus***	12	9.8%	0/5	0/5	4/7	4/8	4/8	0/5	5/5
***Streptococcus gallolyticus* subsp. *gallolyticus***	5	4.1%	0/2	0/2	2/5	1/5	2/5	1/2	2/2
***Streptococcus iniae***	1	0.8%	−	−	−	0/1	0/1	0/1	−
***Streptococcus infantarius* subsp. *coli***	1	0.8%	−	0/1	0/1	0/1	0/1	−	−
***Streptococcus mutans***	1	0.8%	−	−	−	0/1	0/1	0/1	−
***Streptococcus pluranimalium***	1	0.8%	−	−	−	0/1	0/1	0/1	−
***Streptococcus salivarius* subsp. *salivarius***	1	0.8%	−	−	0/1	0/1	0/1	−	−
***Streptococcus thoraltensis***	5	4.1%	0/2	0/2	2/2	1/3	2/3	0/3	2/2
***Streptococcus uberis***	3	2.4%	0/1	0/1	1/2	1/2	1/2	1/1	1/1

CN: gentamicin; SXT: trimethoprim-sulfamethoxazole; P: penicillin; TE: tetracycline; EFT: ceftiofur; ENR: enrofloxacin; DO: doxycycline. −: no result available. ^5^ Two were *Str. dysgalactiae* subspecies *equisimilis*.

**Table 5:** Prevalence of coagulase negative staphylococci isolated in this study and antimicrobial resistance (number of resistant isolates out of the number of tested isolates).

Isolates	Frequency		Antimicrobials Resistance						
	n	%	CN	SXT	P	TE	EFT	ENR	DO
***Staphylococcus capitis***	1	0.8%	0/1	0/1	0/1	0/1	0/1	0/1	0/1
***Staphylococcus chromogenes***	3	2.4%	0/2	0/1	0/2	0/2	0/1	0/1	0/1
***Staphylococcus epidermidis***	2	1.6%	0/1	−	1/1	0/1	−	−	−
***Staphylococcus haemolyticus***	2	1.6%	0/2	0/2	0/2	0/2	1/2	0/2	1/2
***Staphylococcus hyicus***	1	0.8%	0/1	0/1	0/1	0/1	1/1	0/1	0/1
***Staphylococcus saprophyticus***	1	0.8%	−	0/1	0/1	0/1	0/1	0/1	1/1
***Staphylococcus vitulinus***	1	0.8%	−	−	−	−	−	−	−
***Staphylococcus xylosus***	4	3.3%	0/4	0/4	0/4	2/4	0/4	0/4	4/4

CN: gentamicin; SXT: trimethoprim-sulfamethoxazole; P: penicillin; TE: tetracycline; EFT: ceftiofur; ENR: enrofloxacin; DO: doxycycline. −: no result available.

## Data Availability

Dataset available on request from the authors.
